# Prevalence of Intestinal Parasitic Infection among Drug Addicts in District Swat, Khyber Pakhtunkhwa, Pakistan

**Published:** 2019

**Authors:** Wali KHAN, Naveed Iqbal KHAN, Sayed Nimra Faryal BUKHARI, Nishad BEGUM

**Affiliations:** 1. Department of Zoology, University of Malakand, Chakdara, Khyber Pakhtunkhwa, Pakistan; 2. Department of Zoology, University of Hazara, Mansehra, Khyber Pakhtunkhwa, Pakistan

**Dear Editor-in-Chief**

***I***ntestinal parasitic infections are still one of the major health problems in developing countries of the world including Pakistan. Several intestinal parasitic diseases e.g. amebiasis and giardiasis (caused by intestinal protozoa) or schistosomiasis and soil transmitted helminthiasis (caused by parasitic worms) have been categorized as neglected tropical diseases (NTDs), as they continue in poor communities of economical and social importance (1). Intestinal amebiasis caused by *E. histolytica* led to 11,300 deaths worldwide and as ranked 4^Th^ in the most fatal parasite-related diseases in 2013 (2, 3). Soil-transmitted helminths were estimated to infect more than one billion people in 2010 with highest prevalence rates observed in school-aged children (4). Most research on parasitic diseases and related morbidity focuses on single species infections. To date, there are no estimates for drug addicts in a targeted population, on the global burden of diseases due to polyparasitism of intestinal parasitic infections caused by helminths and intestinal protozoa.

In this study, stool samples were collected from 450 drug addicts (150 each from snuffers, smokers and marijuana) in district Swat, Pakistan during 2017.

Each sample was examined by direct wet mount techniques using both normal saline and Lugol’s iodine preparation and concentration procedures using salt and formal–ether solutions. Of the total examined drug addicts only 22.8 % (n=103/450) were found infected with single and or multiple parasite species. Pattern of infection revealed that 90 (87.3%) individuals have single, 8 (7.75%) double and 5 (4.85%) triple infection (Table 1). The prevalence of infections in marijuana addicts was higher 42.7% (n=44) than smokers 31.0% (n=32) and snuffers addicts 26.2% (n=27) respectively. No significant difference was found among the groups (*P*=0.9948). The individuals have age ranging limit was from 15 to 70 year (Table 2).

The study was approved by Ethical Review Committee Department of Zoology, University of Malakand and each of the drug addicts was informed on the merit and demerit of the study.

Intestinal parasitic infections are endemic and have been described as the greatest cause of illness and disease in the world (5). These infections are usually associated with poor hygiene, lack of access to safe water and illiteracy.

**Table 1:** Proportion of mono-parasitism and poly-parasitism of intestinal parasitic infection among drugs addict (snuffers, smokers and marijuana) in district Swat KP, Pakistan

***Type of infection***	***Number of host infected***	***Prevalence (%)***
Mono-parasitism	90	87.3
Hookworm	52	50.4
*Taenia saginata*	10	9.70
*Trichuris trichiura*	9	8.73
*Ascaris lumbricoides*	8	7.76
*Entamoeba histolytica/dispar*	11	10.6
Polyparasitism	13	12.6
With 2 species	8	7.75
*T. saginta +T. trichura*	2	1.94
Hookworm+ *histolytica/dispar*	1	0.97
Hookworm+*T. trichura*	1	0.97
Hookworm +*Ascaris lumbricoides*	4	3.87
With 3 species	5	4.85
*T. trichura+A. lumbricoides+ E. histolytica/dispar*	1	0.97
Hookworm + *T. trichura+ E. histolytica/dispar*	2	1.94
*T. saginta+T. trichura+ E. histolytica/dispar*	1	0.97
*T. saginta+T. trichura+ A. lumbricoides*	1	0.97
Total infected individuals	103	22.8
Total sample tested	450	450

**Table 2:** Prevalence of intestinal parasitic infection in relation to the type of drug addict and ages of the respondents

***Factor***	***No. examined***	***No. infected***	***Prevalence***	***F test***	***R^2^***	***P-value***
**Levels**						
Snuffers	150	27	26.2	0.005180	0.001724	0.9948
Smokers	150	32	31.0			
Marijuana	150	44	42.7			
**Ages (yr)**						
15–30	254	56	54.3	0.1969	0.2598	0.1969
31–45	161	40	38.8			
46–60	29	5	4.8			
61–70	6	2	1.9			

The degree of each factor and the prevalence of infections vary from one region to the other (6). The knowledge of intestinal parasitic infection in a community is important for planning an efficient control programs. Present study assessed the prevalence of intestinal parasitic infection in drug addicts in Swat district Pakistan. A wide variation in the prevalence of intestinal parasite infection was noticed by comparing our result to those already reported studies in many regions of Pakistan. In Pakistan, previous stool surveys have indicated that approximately 12.4%–83.1% of the various studied groups were infected with intestinal parasites (7–8). This large discrepancy in the prevalence of intestinal parasite could mostly be due to the group of studied population. The literature concerned the prevalence of intestinal parasites have mostly been limited to targeted localities and populations in Pakistan.

This paper describes prevalence of intestinal parasites in drug addicts (snuffers, smokers and marijuana) in human populations of Swat, Pakistan. No such studies have been previously been conducted in Pakistan.

## References

[1] Utzinger J Becker SL Knopp S Neglected tropical diseases: diagnosis, clinical management, treatment and control. Swiss Med Wkly. 2012; 142:w13727. 23180107 10.4414/smw.2012.13727

[2] Stanley JR Amoebiasis. Lancet. 2003; 361:1025–34. 12660071 10.1016/S0140-6736(03)12830-9

[3] Vos T Barber RM Bell B Global, regional, and national incidence, prevalence, and years lived with disability for 301 acute and chronic diseases and injuries in 188countries, 1990–2013: a systematic analysis for the Global Burden of Disease Study 2013. Lancet. 2015; 386: 743–800. 26063472 10.1016/S0140-6736(15)60692-4 PMC4561509

[4] Pullan RL Smith JL Jasrasaria R Brooker SJ Global numbers of infection and disease burden of soil transmitted helminth infections in 2010. Parasit Vectors. 2014; 7:37. 24447578 10.1186/1756-3305-7-37 PMC3905661

[5] Keiser J Utzinger J The drugs we have and the drugs we need against major helminth infections. Adv Parasitol. 2010; 73: 197–230. 20627144 10.1016/S0065-308X(10)73008-6

[6] Zaglool DA Khodari YA Othman RAM Farooq MU Prevalence of intestinal parasites and bacteria among food handlers in a tertiary care hospital. Niger Med J. 2011; 52: 266–270. 22529512 10.4103/0300-1652.93802 PMC3329099

[7] Arshad S Khatoon N Warind JA Khan A Waheed S Khan W The prevalence of human intestinal protozoal and helminthic infection in Karachi. Int J Biol Biotech. 2019; 16 (2): 319–323.

[8] Khan W Noor-un-Nisa Khan A Prevalence and Risk Factors Associated with Intestinal Parasitic Infections among Food Handlers of Swat, Khyber Pakhtunkhwa, Pakistan. J Food Nutr Res. 2017; 5(2): 331–336

